# Urine caffeine metabolites and hearing threshold shifts in US adults: a cross-sectional study

**DOI:** 10.1038/s41598-021-01094-9

**Published:** 2021-11-03

**Authors:** Lili Long, Yuedi Tang

**Affiliations:** 1 Department of Otorhinolaryngology, Sichuan University Hospital of Sichuan University, Chengdu, Sichuan China; 2 Department of Otorhinolaryngology Head and Neck Surgery, West China Hospital of Sichuan University, No. 37 Guo-Xue-Xiang, Chengdu, Sichuan China

**Keywords:** Health care, Risk factors

## Abstract

Previous studies have reported the relationship between effect of caffeine and many diseases. However, studies to evaluate the association between caffeine and hearing loss are contradictory. To examine the relationship of urinary caffeine metabolites with the hearing threshold in US adults, a total of 849 adults aged 20–69 years who participated in the National Health and Nutrition Examination Survey (NHANES, 2011–2012) were enrolled in this study. Urinary caffeine and its 14 metabolites were applied as biomarkers to assess caffeine exposure. Hearing loss was defined as mean pure tone averages > 25 dB HL at 500, 1000, and 2000 Hz in both ears (low frequency); and 3000, 4000, and 6000 Hz in both ears (high frequency). Univariate and multivariate linear regression analyses were conducted to examine the associations of urinary caffeine metabolites with low- and high-frequency hearing thresholds, respectively. Low-frequency hearing loss were 5.08% and 6.10% in male and female participants, respectively; and high-frequency hearing loss were 31.81% and 15.14% in male and female participants, respectively. In the unadjusted model, the *P* value for trend shows that urinary caffeine metabolites 137X and AAMU were significantly associated with low-frequency PTA, and that 17X, 137X, AAMU were significantly associated with high-frequency PTA, but when the model was adjusted for sex, age, education level, firearm noise exposure, occupational noise exposure, recreational noise exposure, serum cotinine, body mass index, diabetes, hypertension, these were no longer statistically significant. In conclusion, urinary caffeine metabolites were not associated with the hearing threshold shifts in US adults.

## Introduction

Caffeine is contained widely in dietary consumption of beverages (e.g., coffee, tea, cola drinks) and foods (e.g., chocolate). Caffeine is also used as a food additive in beverages (e.g., caffeinated soft drinks) and as a drug (e.g., in analgesics). As a psychoactive stimulant, its high prevalence in the worldwide diet has developed significant scientific interest in its effects on human health. Studies have reported the relationship between effect of caffeine and many diseases, like diabetes, hyperlipidemia, stroke, heart failure and coronary artery disease^1^. However, studies investigating the relationship between caffeine and hearing loss are contradictory. Two studies reported lower prevalence of hearing loss in coffee consumers^2,3^. Another study reported that chocolate-based diet may protect middle-aged people from hearing loss^4^. Some studies based on animal models suggested that administration of caffeine can exacerbate cisplatin-induced hearing loss and impair the recovery of hearing after an acoustic over stimulation events (AOSE)^5,6^. To date, no large-scale retrospective study has evaluated the relationship between caffeine and hearing threshold shifts among US population.

Assessment of caffeine exposure in epidemiologic studies is conducted mostly by using dietary intake data. However, estimating the caffeine content of dietary sources can be challenging^7^. As the source of most estimates of caffeine intake, food frequency questionnaires or 24-h dietary recalls are usually limited to beverages and do not contain the full caffeine-containing products. Furthermore, the differences in caffeine metabolism among individuals may result in different amounts of circulating caffeine and its metabolites. Testing of urinary caffeine metabolites excretion has been a simple but common way to determine the metabolism and effect of caffeine. In this study, the association of urinary caffeine and 14 caffeine metabolites were determined with hearing threshold shifts in US adults.

## Results

### Baseline characteristics of study participants

Table 1 shows study participants (n = 849) aged between 20 and 69 years (weighted mean, 43.06 ± 14.04 years) including 427 females (weighted, 50.29%) and 422 males (weighted, 49.71%). The means ± standard deviation (SD) of low-frequency and high-frequency pure tone averages (PTA) hearing thresholds in male subjects were 9.56 ± 8.25 and 21.61 ± 17.38, respectively. The hearing thresholds were 9.43 ± 8.28 and 14.23 ± 11.11 in female subjects, respectively. Overall, low-frequency hearing loss were 5.08% and 6.10% in male and female participants, respectively; and high-frequency hearing loss were 31.81% and 15.14% in male and female participants, respectively. There were no statistical differences between male and female participants in low-frequency PTA, race/ethnicity, education level, ratio of family income to poverty (PIR), diabetes status, hypertension status and body mass index (BMI) (all *P* > 0.01).

**Table1:** The weighted demographic characteristics of study participants.

Characteristics	Male	Female	*P* value
Age (years)	41.27 ± 13.53	44.20 ± 13.85	0.0020
BMI (kg/m^2^)	28.71 ± 5.69	28.98 ± 6.88	0.5383
Low-frequency PTA (dB)^a^	9.56 ± 8.25	9.43 ± 8.28	0.8138
High-frequency PTA (dB)^b^	21.61 ± 17.38	14.23 ± 11.11	< 0.0001
**Race/ethnicity (%)**			0.0502
Mexican American	7.53	8.18	
Non-Hispanic White	62.61	68.98	
Non-Hispanic Black	10.97	10.74	
Other races	18.89	12.10	
**Education level (%)**			0.0654
Below high school	16.19	12.88	
High school	20.32	16.10	
Above high school	63.49	71.01	
**PIR (%)**			0.6766
< 1	19.02	15.81	
≥ 1, < 5	54.29	56.59	
≥ 5	21.79	22.64	
Diabetes (%)	7.06	7.11	0.9022
Hypertension (%)	28.86	25.52	0.3979
Serum cotinine (≥ 10 ng/mL) (%)	31.30	18.47	< 0.0001
Firearm noise exposure (%)	58.44	27.96	< 0.0001
Occupational noise exposure (%)	43.63	22.44	< 0.0001
Recreational noise exposure (%)	18.75	10.05	0.0003
Low-frequency hearing loss (%)	5.08	6.10	0.5180
High-frequency hearing loss (%)	31.81	15.14	< 0.0001

Values are weighted means ± SD for: age, BMI, low-frequency PTA, high-frequency PTA. *P* value was calculated by weighted linear regression model. % for: race/ethnicity, education level, PIR, diabetes, hypertension, serum cotinine, firearm noise exposure, occupational noise exposure, recreational noise exposure, low-frequency hearing loss, high-frequency hearing loss. P value was calculated by weighted chi-square test. Created by EmpowerStats (www.empowerstats.com) and R.

^a^Mean PTA at 0.5, 1, and 2 kHz of both ears.

^b^Mean PTA at 3, 4, 6 kHz of both ears.

### The univariate analysis of comparison of variables in low-frequency and high-frequency PTA groups

Table 2 shows age, education level, occupational noise exposure, recreational noise exposure, cotinine, diabetes, hypertension, urinary 1,3,7 trimethyluric acid (137U), 1,7-dimethylxanthine (paraxanthine) (17X) and 1,3,7-trimethylxanthine (caffeine) (137X) had a significant univariate correlation (*P* ≤ 0.01) with low-frequency PTA; and age, sex, education level, firearm noise exposure, occupational noise exposure, recreational noise exposure, BMI, diabetes, hypertension, urinary caffeine metabolites 3-methyluric acid (3U), 7-methyluric acid (7U), 17X, 137X and 5 acetylamino-6-amino-3-methyluracil (AAMU) had a significant univariate correlation (*P* ≤ 0.01) with high-frequency PTA. Race, PIR and urinary caffeine metabolites 1-methyluric acid (1U), 1,3 dimethyluric acid (13U), 1,7-dimethyluric acid (17U), 3,7-dimethyluric acid (37U), 1-methylxanthine (1X), 3-methylxanthine (3X), 7-methylxanthine (7X), 1,3 dimethylxanthine (theophylline) (13X) and 3,7 dimethylxanthine (theobromine) (37X) had no correlation with either low- or high-frequency PTA hearing threshold shifts (shown in Supplementary Table S1).

**Table 2:** The univariate analysis of comparison of variables in low-frequency and high-frequency PTA groups.

Variable	N (%)/Mean ± SD	Low-frequency PTA		High-frequency PTA	
		β (95% CI)	*P* value	β (95% CI)	*P* value
Sex (female)	427 (50.29%)	− 0.13 (− 1.25, 0.98)	0.8138	− 7.38 (− 9.32, − 5.44)	< 0.0001
Age (years)	43.06 ± 14.04	0.25 (0.21, 0.29)	< 0.0001	0.58 (0.51, 0.64)	< 0.0001
BMI (kg/m^2^)	28.99 ± 6.94	0.05 (− 0.03, 0.14)	0.2143	0.21 (0.06, 0.37)	0.0073
Education level (above high school)	521 (61.37%)	− 3.63 (− 5.19, − 2.07)	< 0.0001	− 7.33 (− 10.15, − 4.51)	< 0.0001
Firearm noise exposure	297 (34.98%)	0.04 (− 1.08, 1.17)	0.9388	3.64 (1.63, 5.64)	0.0004
Occupational noise exposure	278 (32.74%)	1.70 (0.51, 2.88)	0.0052	5.27 (3.16, 7.37)	< 0.0001
Recreational noise exposure	105 (12.37%)	2.19 (0.59, 3.78)	0.0073	9.27 (6.47, 12.07)	< 0.0001
Cotinine (≥ 10 ng/mL)	205 (24.15%)	2.18 (0.88, 3.47)	0.0010	3.06 (0.73, 5.38)	0.0101
Diabetes	94 (11.07%)	3.14 (0.99, 5.30)	0.0044	8.38 (4.53, 12.23)	< 0.0001
Hypertension	262 (30.86%)	2.16 (0.92, 3.41)	0.0007	6.36 (4.16, 8.57)	< 0.0001
3U (umol/L)	0.95 ± 2.27	0.11 (− 0.12, 0.34)	0.3524	0.55 (0.14, 0.95)	0.0089
7U (umol/L)	23.20 ± 41.96	0.01 (− 0.00, 0.02)	0.0689	0.04 (0.01, 0.06)	0.0019
137U (umol/L)	2.91 ± 4.08	0.17 (0.04, 0.31)	0.0100	0.26 (0.02, 0.49)	0.0364
17X (umol/L)	27.31 ± 30.28	0.03 (0.01, 0.05)	0.0036	0.07 (0.04, 0.11)	< 0.0001
137X (umol/L)	7.02 ± 9.04	0.10 (0.04, 0.16)	0.0011	0.20 (0.09, 0.31)	0.0003
AAMU (umol/L)	101.60 ± 140.28	0.00 (0.00, 0.01)	0.0105	0.01 (0.01, 0.02)	0.0004

### Association between urinary caffeine metabolite concentrations and hearing thresholds

Table 3 shows the estimated association of urinary caffeine metabolites with low-frequency and high-frequency hearing thresholds using multivariate linear regression model. All the significant variables in univariate analysis were included in this analysis. Urinary 3U, 7U, 137U, 17X, 137X and AAMU were converted to a categorical variable (tertiles), and were used as a continuous variable to calculate the linear trend. In the unadjusted model (crude model), the *P* value for trend shows that 137X and AAMU were significantly associated with low-frequency PTA, and that 17X, 137X, AAMU were significantly associated with high-frequency PTA. In the fully adjusted model (model 2), the *P* value for trend shows there was no significant linear trend among tertiles of urinary 3U, 7U, 137U, 17X, 137X and AAMU and both low-frequency and high-frequency PTA hearing thresholds (all *P* > 0.01).

**Table 3:** Association between urinary caffeine metabolites and hearing threshold at low and high frequencies.

Variables (umol/L)	Low-frequency PTA (dB) β (95% CI) *P*_trend_			High-frequency PTA (dB) β (95% CI) *P*_trend_		
	Crude Model	Model 1	Model 2	Crude Model	Model 1	Model 2
3U	0.13 (− 0.56, 0.82)	− 0.43 (− 1.06, 0.20)	− 0.17 (− 0.78, 0.44)	1.01 (− 0.23, 2.25)	0.07 (− 0.92, 1.06)	0.02 (− 0.96, 1.00)
	0.7149	0.1839	0.5892	0.1107	0.8867	0.9680
7U	0.17 (− 0.51, 0.85)	− 0.16 (− 0.78, 0.46)	0.01 (− 0.58, 0.61)	0.78 (− 0.44, 2.00)	0.32 (− 0.65, 1.28)	0.14 (− 0.81, 1.09)
	0.6202	0.6058	0.9662	0.2101	0.5230	0.7742
137U	1.63 (0.36, 2.90)	0.36 (− 0.30, 1.01)	0.75 (0.12, 1.37)	1.63 (0.36, 2.90)	0.56 (− 0.46, 1.58)	0.78 (− 0.22, 1.77)
	0.0120	0.2878	0.0192	0.0120	0.2815	0.1253
17X	0.47 (− 0.22, 1.16)	− 0.13 (− 0.75, 0.49)	− 0.05 (− 0.66, 0.56)	1.87 (0.65, 3.09)	0.59 (− 0.38, 1.57)	0.68 (− 0.28, 1.65)
	0.1789	0.6807	0.8680	0.0028	0.2344	0.1630
137X	1.05 (0.36, 1.73)	− 0.06 (− 0.71, 0.59)	0.34 (− 0.28, 0.95)	1.83 (0.61, 3.06)	− 0.34 (− 1.35, 0.67)	0.12 (− 0.86, 1.11)
	0.0028	0.8531	0.2846	0.0035	0.5092	0.8069
AAMU	1.42 (0.73, 2.10)	0.63 (− 0.01, 1.27)	0.47 (− 0.14, 1.08)	3.14 (1.92, 4.37)	1.29 (0.30, 2.28)	0.81 (− 0.17, 1.78)
	< 0.0001	0.0523	0.1348	< 0.0001	0.0108	0.1057

Crude Model = unadjusted. Model 1 = Crude Model + sex, age. Model 2 = Model 1 + education level, firearm noise exposure, occupational noise exposure, recreational noise exposure, cotinine, BMI, diabetes, hypertension.

## Discussion

Caffeine known to have psychoactive stimulatory effects is widely used in the world. It’s in our beverages (e.g., coffee, tea, cola drinks) and foods (e.g., chocolate), as well as in certain medications (e.g., analgesics). Its high prevalence of intake in the worldwide diet has arisen significant scientific interest in its effects on human health. Caffeine consumption has been studied as a risk factor for many diseases and conditions, including hypertension, cardiovascular diseases, bone density, various cancers, mental and behavioral disorders, and reproduction and developmental abnormalities^8–10^. However, studies to evaluate the effect of caffeine on hearing loss are contradictory. Two studies based on test of guinea pigs showed that a daily dose of caffeine can impair the recovery of hearing after an acoustic overstimulation event^6,11^. Another study conducted using a rat model of cisplatin ototoxicity suggested that caffeine consumption may exacerbate cisplatin-induced hearing loss^5^. Contradictorily, two investigations reported lower prevalence of hearing loss in coffee consumers^2,3^. Another study reported the protective effect of chocolate-based diet on hearing loss^4^. This retrospective study has conducted to evaluate the relationship between caffeine and hearing threshold shifts among US population. To our knowledge, this is the first study examining association between urinary caffeine metabolites and hearing threshold shifts in US adults. Regression models were designed to investigate relationships between 15 caffeine metabolites detected in urine samples and PTA at low and high frequency. The findings of this study differ from those previous studies^2,4–6,11^. In the present study, there was no effect of caffeine exposure on hearing threshold shifts.

The results of studies on the effect of caffeine on hearing loss are inconsistent for several reasons. First, caffeine intake from source of food frequency questionnaires or 24-h dietary recalls may be misclassified and underreported, which make assessment of caffeine exposure in epidemiologic studies to be challenging^7,12^. People have been taking efforts to improve the quality of dietary intake data^13^. Studies have shown the possibility of using caffeine and caffeine metabolites as biomarkers of caffeine intake^14–17^. This study shows that 137U, 17X and 137X had a significant univariate correlation (*P* ≤ 0.01) with low-frequency PTA; and that 3U, 7U, 17X, 137X and AAMU had a significant univariate correlation (*P* ≤ 0.01) with high-frequency PTA. These compounds are almost all the ones shown promise as potential biomarkers of caffeine intake in the previous study^14^. Second, it should be noted that besides the challenge in calculating caffeine intake from food, the common food (e.g., coffee and chocolate) for study caffeine contain agents other than caffeine. Coffee has high levels of different polyphenols with antioxidant and anti-inflammatory effects which can protect against oxidative-related disorders, including age-related hearing loss^3,18–20^; polyphenols, phenethyl ester, and other bioactive phytochemicals in coffee have been demonstrated protective effects against ototoxicity in the peripheral auditory system^8,21,22^. However, caffeine was reported to have the opposite effect. Oral administration of caffeine can exacerbate cisplatin-induced hearing loss^5^. Besides those agents, coffee also contains active compounds like trigonelline, which is determined to be the protective agent in coffee for animals with diabetic neuropathy, but not caffeine^23^. Furthermore, in the aspect of analysis, the conclusion of “daily coffee consumers had 50–70% less hearing loss than rare coffee consumers” in the previous study was not been calculated using multivariate model. The protective effect of daily coffee consumption is only on a narrow range of people who were aged between 40 and 69 years old with bilateral hearing loss after multivariate analysis^8^.

The data in this study is from the NHANES which contains a large and nationally representative sample of participants in the US. The measurements of demographics data, audiometry data, laboratory examination data, and questionnaire data in the NHANES are standardized and reliable. Despite these strengths, some limitations of this study should be taken into consideration. The temporal relationship between urinary caffeine metabolites levels and hearing loss is not confirmed for the NHANES is a cross-sectional study. Some potential covariates were not calculated in our models. Only 2 years of the NHANES data (2011–2012) were used, thus, the proportion of the adults who met standards in this study is relatively small and larger cohort studies may be needed to confirm the findings. In conclusion, according to the results of NHANES analyses, urinary caffeine metabolites were not associated with hearing threshold shifts in US adults.

## Methods

### Ethics statement

The NHANES data were approved by the National Center for Health Statistics Institutional Review Board. Informed consents were obtained from all participants before the surveys. All methods were carried out in accordance with the relevant guidelines and regulations of NHANES.

### Design and participants

The NHANES study is an ongoing cross-sectional survey based on a randomly-selected sample of national residents in the United States^24^. It is administered by the institutional Review Board of the National Center for Health Statistics of the Centers for Disease Control and Prevention. The survey combines Initial screening questions to determine qualified participants, extensive interviews held at home and physical examination or clinical evaluations performed at mobile examination centers. Detailed questionnaire instruments, brochures, procedure manuals, and consent documents for the 2011–2012 NHANES are publicly accessible from the NHANES website (https://wwwn.cdc.gov/nchs/nhanes/ContinuousNhanes/Default.aspx?BeginYear=2011).

**Figure 1:**
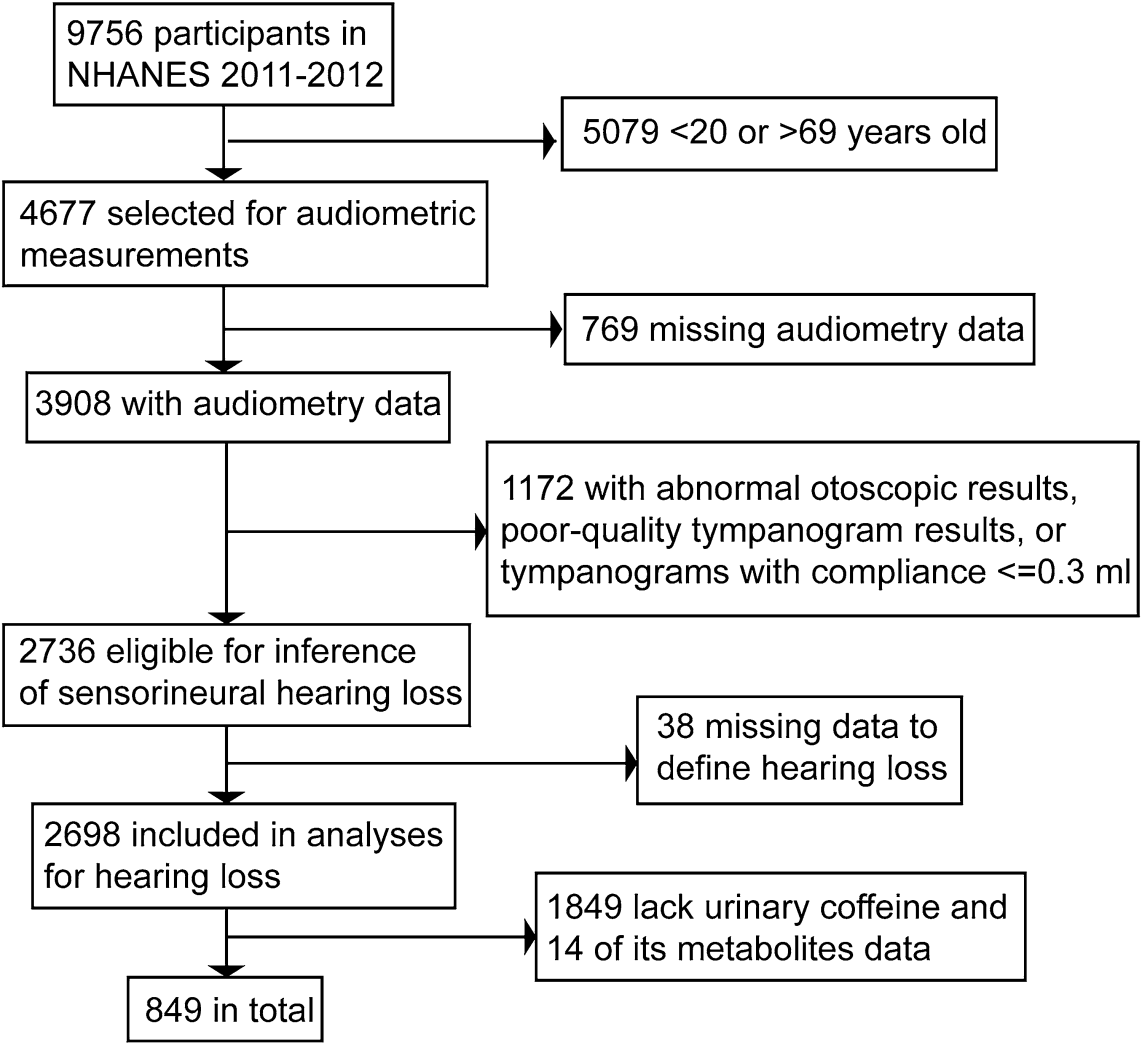
Flow chart of the selection process. *NHANES* national health and nutrition examination survey.

The data from the 2011–2012 cycle of NHANES are used for this retrospective study because these were the only years when both urine caffeine samples and audiometry data of participants aged 20–69 years were collected at the time of study. There were 9756 participants in the NHANES dataset from 2011 to 2012. After excluding those with missing data such as audiometry data, urinary caffeine analysis data, and those with abnormal otoscopic results, poor-quality tympanogram results or tympanograms with compliance ≤ 0.3 mL which indicate the likelihood of conductive hearing loss, 849 participants were included in our analysis. Figure 1 shows the flow chart of participant selection.

### Audiometric measurements

Audiometry data were collected from participants aged 20–69 years old. The ears of participants were examined by a trained examiner using a Welch Allyn otoscope (model 25020). The Earscan Acoustic Impedance tympanometer (Micro Audiometrics) was used for tympanometry which was performed by measuring the sound pressure level of a 226-Hz probe tone introduced into the ear canal at 85 dB while varying the air pressure in the ear canal from 200 to − 312 daPa. An AD226 audiometer, TDH-39P headphones and EARTone 3A earphones were used for audiometry of both ears. Manual testing was used instead of automated audiometric testing when the subject had difficulties in operating the response switch^25^. Participants who were unable to remove hearing aids for examining and testing and participants with ear pain who could not tolerate headphones were excluded.

In this study, low-frequency hearing loss was defined as mean PTA at 500, 1000, and 2000 Hz > 25 dB HL in both ears; and high-frequency hearing loss was defined as mean PTA at 3000, 4000 and 6000 Hz > 25 dB HL in both ears^26–28^.

### Measurement of caffeine metabolites in urine

Urine samples were collected by trained operators at the mobile examination centers and were tested in the National Center for Environmental Health (the Centers for Disease Control and Prevention, Atlanta, GA, USA) using ultra-high performance liquid chromatography–electrospray ionization–tandem quadrupole mass spectrometry (AgilentTechnologies, Palo Alta, CA, USA). Caffeine and 14 of its urinary metabolites, 15 in total, were tested, including 1U, 3U, 7U, 13U, 17U, 37U, 137U, 1X, 3X, 7X, 13X, 17X, 37X, 137X, and AAMU. More detailed procedures are described on the NHANES website^29^.

### Covariates

Potential covariates considered in the analyses included: age as continuous variables; sex, race/ethnicity, education level, PIR, diabetes, hypertension, serum cotinine, BMI, firearm noise exposure, occupational noise exposure and recreational noise exposure as categorical variables. Information on age, sex, race/ ethnicity, education level, PIR, diabetes, hypertension, firearm noise exposure, occupational noise exposure and recreational noise exposure were obtained during in-home interviews. BMI were calculated with data on weight and height measured through physical examination. Serum cotinine level, a marker for both active and passive tobacco exposure, was measured using an isotope dilution-high performance liquid chromatography with an atmospheric pressure chemical ionization tandem mass spectrometry^30^.

Diabetes was defined as “other than during pregnancy, ever been told by a doctor or health professional had diabetes or sugar diabetes”. The answer of “borderline” was also considered as diabetes^28^. Hypertension was defined as “ever been told by a doctor or other health professional had hypertension, also called high blood pressure”^28^. Firearm noise exposure was defined as “ever used firearms for any reason”, occupational noise exposure was defined as “ever had a job, or combination of jobs exposed to loud sounds or noise for 4 or more hours a day, several days a week”, recreational noise exposure was defined as “ever been exposed to very loud noise or music for 10 or more hours a week”^31^.

### Statistical analysis

The 2-year C subsample weights (WTSC2YR) of 2011–2012 survey cycle were used in this study to estimate more representative measures for general population in the United States following the analytic guidelines of NHANES. The baseline characteristics of the study participants were divided into two groups by sex and analyzed (Table 1). The continuous variable was expressed as means ± SD. *P* value was calculated by weighted linear regression model. The categorical variables were expressed as percentages. *P* value was calculated by weighted chi-square test. Potential variables were selected and univariate analysis was done (Table 2 and Supplementary Table S1). All the significant variables with a level of significance at *P* ≤ 0.01 were included in a multivariate linear regression model used to investigate regression coefficients (β) and 95% confidence intervals (CIs) between urinary caffeine metabolites and mean bilateral low-frequency and high-frequency PTA (Table 3). Three models were provided to adjust the effect of relevant covariates: the no adjustment (Crude mode); adjustment for age and sex (Model 1); further adjustment for, education level, firearm noise exposure, occupational noise exposure, recreational noise exposure, cotinine, BMI, diabetes and hypertension (Model 2). The analyses were conducted by statistical programming language R (version 3.6.1, The R Foundation) and EmpowerStats software (X&Y Solutions, Inc.).

## Supplementary Information

Supplementary Table 1.

## Abbreviations

NHANES: The national health and nutrition examination survey
PTA: Pure tone average
PIR: Ratio of family income to poverty
BMI: Body mass index
1U: 1-Methyluric acid
3U: 3-Methyluric acid
7U: 7-Methyluric acid
13U: 1,3 Dimethyluric acid
17U: 1,7-Dimethyluric acid
37U: 3,7-Dimethyluric acid
137U: 1,3,7 Trimethyluric acid
1X: 1-Methylxanthine
3X: 3-Methylxanthine
7X: 7-Methylxanthine
13X: 1,3 Dimethylxanthine
17X: 1,7-Dimethylxanthine
37X: 3,7 Dimethylxanthine
137X: 1,3,7-Trimethylxanthine
AAMU: 5 Acetylamino-6-amino-3-methyluracil
