# Supplementary material for: Urine caffeine metabolites and hearing threshold shifts in US adults: a cross-sectional study
Source: Sci Rep. 2021 Nov 3;11:21631. doi: 10.1038/s41598-021-01094-9 (PMC8566481; doi:10.1038/s41598-021-01094-9)
Supplement: Supplementary file 1 — Supplementary Table 1. [file 41598_2021_1094_MOESM1_ESM.docx]

Supplementary Table 1 The univariate analysis of comparison of variables in low-frequency and high-frequency PTA groups

| variable | | N(%)/ Mean±SD | low-frequency PTA | | high-frequency PTA | |
| --- | --- | --- | --- | --- | --- | --- |
|  |  |  | β (95% CI) | *P* value | β (95% CI) | *P* value |
| Race/Ethnicity | Mexican | 89 (10.48%) | Reference |  | Reference |  |
|  | non-Hispanic white | 304 (35.81%) | -0.23 (-2.33, 1.87) | 0.8275 | 1.40 (-2.35, 5.15) | 0.4641 |
|  | non-Hispanic black | 217 (25.56%) | -0.12 (-2.72, 2.49) | 0.9311 | -1.68 (-6.34, 2.98) | 0.4802 |
|  | other races | 239 (28.15%) | 0.58 (-1.86, 3.03) | 0.6392 | 2.94 (-1.43, 7.31) | 0.1879 |
| PIR | <1 | 205 (24.15%) | Reference |  | Reference |  |
|  | ≥1, <5 | 443 (52.18%) | -0.95 (-2.48, 0.58) | 0.2226 | 1.70 (-1.05, 4.45) | 0.2255 |
|  | ≥5 | 142 (16.73%) | -1.88 (-3.66, -0.10) | 0.0386 | 1.92 (-1.28, 5.12) | 0.2388 |
| Urinary caffeine metabolites | 1U (umol/L) | 99.11 ± 157.99 | 0.00 (-0.00, 0.01) | 0.2052 | 0.01 (-0.00, 0.01) | 0.0519 |
|  | 13U (umol/L) | 13.41 ± 77.09 | -0.00 (-0.01, 0.00) | 0.3903 | -0.00 (-0.01, 0.01) | 0.9235 |
|  | 17U (umol/L) | 45.66 ± 62.57 | 0.01 (0.00, 0.02) | 0.0188 | 0.02 (0.00, 0.03) | 0.0113 |
|  | 37U (umol/L) | 1.69 ± 2.91 | -0.04 (-0.23, 0.15) | 0.6520 | 0.03 (-0.31, 0.38) | 0.8421 |
|  | 1X (umol/L) | 54.57 ± 84.87 | 0.00 (-0.00, 0.01) | 0.2275 | 0.01 (-0.01, 0.02) | 0.2487 |
|  | 3X (umol/L) | 46.02 ± 79.92 | -0.00 (-0.01, 0.00) | 0.7847 | 0.00 (-0.01, 0.01) | 0.5470 |
|  | 7X (umol/L) | 75.35 ± 115.11 | -0.00 (-0.01, 0.00) | 0.7019 | 0.00 (-0.01, 0.01) | 0.5854 |
|  | 13X (umol/L) | 3.43 ± 20.16 | -0.01 (-0.02, 0.01) | 0.3421 | -0.00 (-0.03, 0.03) | 0.8376 |
|  | 37X (umol/L) | 27.86 ± 40.27 | -0.00 (-0.02, 0.01) | 0.7797 | 0.00 (-0.02, 0.03) | 0.7171 |
